# Hyaluronic Acid: Perspectives in Upper Aero-Digestive Tract. A Systematic Review

**DOI:** 10.1371/journal.pone.0130637

**Published:** 2015-06-29

**Authors:** Manuele Casale, Antonio Moffa, Lorenzo Sabatino, Annalisa Pace, Giuseppe Oliveto, Massimiliano Vitali, Peter Baptista, Fabrizio Salvinelli

**Affiliations:** 1 Area of Otolaringology, University Campus Bio-Medico, Via Alvaro del Portillo 21, Rome, Italy; 2 Bio-Statistical Department, University Campus Bio-Medico, Via Alvaro del Portillo 21, Rome, Italy; 3 Department of Otolaringology, University of Navarra, Campus Universitario, Pamplona, Spain; University of Insubria, ITALY

## Abstract

**Background:**

To date, topical therapies guarantee a better delivery of high concentrations of pharmacologic agents to the mucosa of the upper aerodigestive tract (UADT). The use of topical drugs, which are able to reduce mucosal inflammation and to improve healing tissues, can represent a relevant therapeutic advance. Topical sodium hyaluronate (SH) has recently been recognized as adjuvant treatment in the chronic inflammatory disease of the UADT.

**Aims:**

The aim of our work was to review the published literature regarding all the potential therapeutic effects of SH in the chronic inflammatory disease of UADT.

**Methods:**

Relevant published studies were searched in Pubmed, Google Scholar, Ovid using keywords (“sodium hyaluronate” and “upper airways”) or Medical Subject Headings.

**Results:**

At the end of our selection process, sixteen publications have been included. Six of them in the post-operative period of nasal-sinus surgery, 2 of them in pediatric patients affected by recurrent upper respiratory tract infections, 4 of them in reducing symptoms and preventing exacerbations of chronic upper airways in adult population, 4 of them in patients with chronic inflammatory disease of UADT, including gastro-esophageal reflux disease (GERD).

**Conclusions:**

Topical administration of SH plays a pivotkey role in the postoperative phase of patients undergoing FESS and nasal surgery, and positive results are generally observed in all the patients suffering from UADT chronic inflammatory disease.

## Introduction

Hyaluronan is a naturally occurring polysaccharide which consists of a linear chain of fragments of d-glucuronic acid and *N*-acetyl-glucosamine linked by alternating β-3 and-β4 bonds. It is an abundant constituent of the extracellular matrix of connective tissue, synovial fluid, embryonic mesenchyme, vitreous humor, skin, and several other organs and tissues of the human body [1]. Hyaluronan may be an important regulator of inflammatory response: high molecular weight hyaluronan is broken down under the influence of free radicals and enzymes during inflammation. Low molecular weight fragments deliver signal about tissue damage and mobilize immune cells, while high molecular weight form suppresses immune response preventing from excessive exacerbations of inflammation [2].

Depending on molecular weight, hyaluronan can have different effects.

Low molecular weight hyaluronan (<300 kD) stimulates cell proliferation and initiates pathways involving inflammation and stimulates ciliary beat frequency (CBF) via RHAMM (receptor for hyaluronic acid mediated motility); instead, high molecular weight hyaluronan (1,000 kD) has no effect on CBF.

Airway’s epithelium synthesizes high-molecular-weight hyaluronan, that needs to be degradated by oxidative stress (ROS, reactive oxygen species) induced by allergen challenge in airway lumen in order to signal via RHAMM. The low molecular weight fragments of hyaluronan such generated trigger RHAMM and RON (recepteure d’origine nantais) pathways and increase CBF [2].

CD44 is another crucial HA receptor. It is found on many cell types involved in inflammation, including leukocytes, chondrocytes, fibroblasts, and endothelial and epithelial cells and has been implicated in various processes such as lymphocyte recruitment. Its bound with HA is mediator of different signals depending on molecular weight in a similar manner of RHAMM/RON pathways. In fact low molecular weight HA is shown to stimulate proliferation, whereas high molecular weight fractions were inhibitory. The precise mechanism remains unclear [3].

TLR in another low molecular weight HA protein involved in inflammation signaling. There is no evidence of direct physical binding between TLR4 and HA, but is undoubtable that the both play a role in the same pathway. Its signaling can be CD44 both independent and dependent. In dependent one, it is unclear if CD44 plays a role as competitive or co-operative way [4].

Hyaluronan is also present on the airway surface, being an important component of normal airway secretions, and certainly playing a central role in the homeostasis of physiology in the respiratory apparatus, especially at level of the upper airway [3]. In particular, in the nasal mucosa hyaluronan is primarly involved in the regulation of vasomotor tone and gland secretion and it significantly contributes to mucosal host defense by stimulating ciliary clearance of foreign bodies while simultaneously retaining enzymes which are important for homeostasis in the apical surface [3]. Hyaluronan, which is an hygroscopic macromolecule and its solutions are highly osmotic, forms a scaffold that several sulfur proteoglycans bind to. Such structures can reach large size and are able to trap large quantities of water and ions, providing hydration and tissue turgescence. Data from some studies pointed out that in the oral mucosa this property either enables the control of tissue hydration during inflammation process or it allows the response to tissue injury which results in ulcer formation. As its structure doesn’t exhibit species specificity or tissue specificity, its pure form doesn’t have any allergizing or immunogenic properties [4,5]

Up to date, SH is widely used in several other branches of medicine and neither contraindications nor interactions with drugs are reported [6,7,8,9].

Its very extensive use in the world is a consequence of its high level of safety. SH is widely used in esthetic medicine as a filling material for folds and creases and to enlarge some parts of human body (such as lips, breasts, buttocks, etc.), it is also employed for the treatment of arthritis [7] and a lot of studies on the effect of SH in the lung disease are available in literature, as well [10]. Formulations of SH have been developed for topical administration as coadjutant treatment in clinical cases of acute and chronic pathologies in the upper aero digestive tract (UADT) and in the tissue healing after UADT surgery, based on the large evidence of data available on SH role in UADT, in animals models [11,12]

The aim of our work is to systematically review the published literature regarding all the potential therapeutic effects of SH in the chronic inflammatory disease of UADT, treating to better investigate the main application areas of SH in UADT, the root and schedule of SH administration, the main efficacy parameters evaluation of SH in UADT.

## Methods

### Search and study selection

Relevant published studies were searched in Pubmed, Google Scholar, Ovid, using either the following keywords or, in case of Pubmed database, Medical Subject Headings: (“sodium hyaluronate” and “upper airways”), (“sodium hyaluronate” and “chronic rhinosinusitis”), (“sodium hyaluronate” and “rhinitis”) and (“sodium hyaluronate” and “turbinate hypertrophy”) (“sodium hyaluronate” and “gerd”), (“sodium hyaluronate” and “oral ulcers”) (“sodium hyaluronate” and “larynx”), (“sodium hyaluronate” and “tonsils”), (“sodium hyaluronate” and “pharyngitis”) with no limit for the year of publication.

Only studies in English, published in peer-reviewed journals, reporting data on the role of the topical administration of sodium hyaluronate in the UADT, in the oral ulcers and mucositis, were included. No studies related to dentistry have been considered.

Literature reviews, technical notes, letters to editors, and instructional course were excluded.

Two authors (CM and MA) independently assessed the full-text version of each publication, by selecting that on the basis of its content and excluding papers without the specific content. The reference lists of the selected articles were fully and accurately reviewed to identify articles not included during the first electronic search.

Taking into account all the journals, articles strictly coherent with the topic were firstly identified, while excluding studies on animal models after a primary selection.

Studies that were based on the use of SH resorbable nasal packing or dressing were not included either. Furthermore, any scientific papers regarding invasive administration of hyaluronic acid, with the exception for those related to the Empty Nose Syndrome, were not considered for the aims of our research, as shown in S1 List of Excluded Studies.

**Fig 1:**
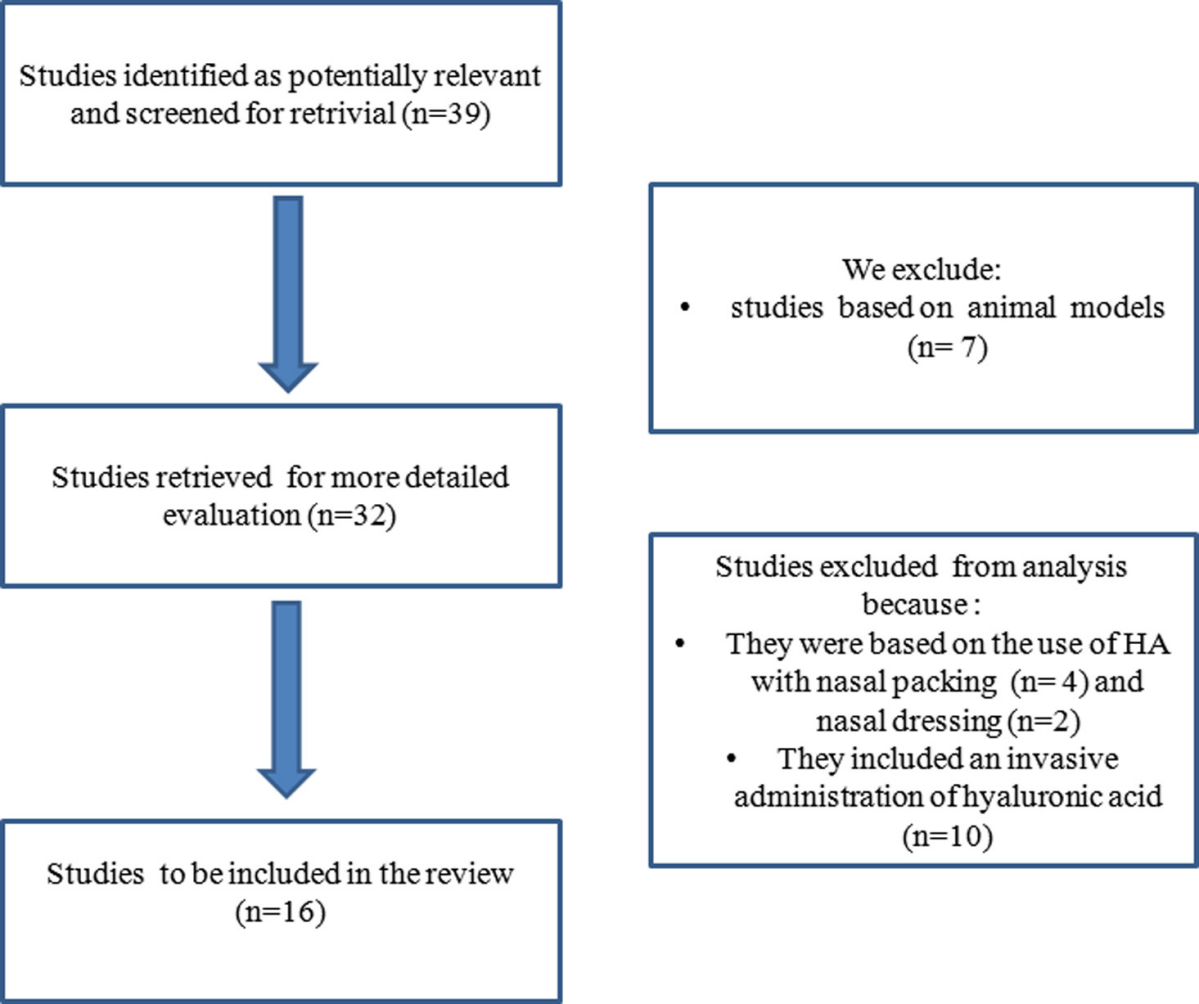
Flow chart of articles research for systematic review.

At the end of our study selection process, 16 relevant publications have been included as shown in Fig 1.

## Results

Sixteen clinical studies, which investigated the role of SH, have been included in the present review, as below reported. Six of them in the post-operative period of nasal-sinus surgery, 2 of them in pediatric patients affected by recurrent upper respiratory tract infections, 4 of them in reducing symptoms and preventing exacerbations of chronic upper airways in adult population, 4 of them in patients with chronic inflammatory disease of UADT, including gastro-esophageal reflux disease (GERD). Study design and sample size of each single study are reported in Table 1.

**Table 1:** Included studies.

Authors	Study design	Patients distribution	Mean age (years)	Disease	Therapy	Efficacy parameters	Results	Side effects
Macchi A. et al. [13]	Prospective, open label, rnd, controlled, parallel group	46 patients: SH group (23); Control group (23)	SH group (37); Control grou**p** (40)	Patients affected by chronic sinusitis undergoing FESS	Eligible patients started aerosol therapy with nasal washes. Intermittent treatment was administered twice daily for 15 consecutive days per month, for three consecutive months. The patients in the SH group receive 9 mg vial of sodium hyaluronate plus 3 ml saline, or 6 ml saline alone (control group).	Endoscopic evaluati**o**ns: (1) improvment of endoscopic parameters (opening of paranasal sinuses, oedema, respiratory patency, sinechiae; (2) appearence of mucosa (3) appearence of nasal secretions. Cytology:(1) ciliary motility (2) neutrophils (3) eosinophils (4) mast cells (5)bacteria (6) mycetes	Intermittent treatment with SH solution was associated with significant improvements in terms of endoscopic appearance (reduction of nasal edema, crusting and secretions). Nasal secretions in control group were more catarrhal, purulent or hematic than in the SH group. Significant differences which favoured SH group, were observed for ciliary motility and presence of mycetes.	
Casale M, et al [14]	Prospective,open label, rnd, controlled, parallel group	57 Patients: SH group(22) Control group (35)	SH group (42) Control group (48)	Patients affected by chronic inferior turbinate hypertrophy undergoing radiofrequency for turbinate volume reduction (RFTVR)	SH group received SH (3 ml of SH is dissolved in 2 ml of isotonic solution) twice a day through Rinowash. Ccntrol group received standard saline nasal irrigation twice a day. The treatment in both groups was applied from the 1st postoperative day and continued during 14 days.	Nasal subjective symptoms were graded using a VAS. Endoscopic nasal score	Mean VAS score of the SH group at week 1 was significantly lower than control group. VAS score remained significantly lower in SH group also at week 2, becoming similar to the control group at week 4. SH group showed lower endoseopic nasal scores (statistically significant in the first 2 weeks) than control group, especially for crusts.	
Gelardi M. et al [15]	Prospective,blinded, rnd, controlled, parallel group	36 patients: SH group(19); Control group (17)	SH group (47); Control group(47)	Patients affected by grade II nasal polyposis undergoing FESS.	SH group was treated with SH (9mg nebulized in 3mL sodium chloride 0.9%) twice a day. Control group was treated with 5mL sodium chloride 0.9% twice a day. After surgery patients were treated for 30 days starting on the second day after surgery.	Clinical outcomes: burning, dryness, nasal obstruction, rhinorrhea. Endoscopic outcome: exudate. Functional outcome: t MCT time to mucous ciliary clearance.		
Cantone E. et al [16]	Prospective,double blind, rnd, controlled, parallel group	122 Patients: SH group (62); Control group (60)	SH group (41); Control group (42)	Patients affected by grade II nasal polyposis undergoing to FESS	SH group was treated with intranasal administration of 9mg SH nebulized in 2 ml sodium chloride 0,9%. Control group was treated with 5 mL sodium chloride 0.9% alone. In both groups, treatments were administered twice a day for 30 consecutive days starting on the first day after surgery.	The following test were used for the assessment of QoL in CRS patients: Short Form-36 (SF-36) test, Sino-Nasal Outcome Test-22 (SNOT-22),Visual Analogue Scale (VAS). Endoscopic score.	After postoperative treatment, the endoscopic score, the total VAS score, the mean SNOT-22 sum score, and SF-36 results were better in SH group than in control group.	
Soldati D. et al.[17]	Prospective,open label, rnd, controlled, parallel group	56 Patients: SH group (27); Control group (29)	SH (33); Ccontrol group (33)	56 patients undergoing FESS	The patients were recruited 2 days after surgery. The patients in both group were instructed to apply the product in a sufficient amount on the nasal mucosa and to make it penetratethe wings of the nose by gently massage. SH group was treated with SH creamgroup (hyaluronic acid in the form of 0,2% nasal cream Rhinogen) while control group was treated with (nasal ointment H.E.C). The apllication were repeated 3–4 times a day during the whole duration of the study period (42 days).	Mucosa dryness, crusting, presence of blood crusts and lesion of nasal mucosa were assessed by the investigators; mucosa dryness, crusting, nasal breathing, tolerability and organoleptic parameters were assessed by the patients.	With regard to nasal breathing patiens in the SH cream group showed a faster and greater improvement than those in the control group. Furthermore, SH cream prevented extensive crust formation during the first week of wound healing. The analysis of efficacy, showed the overall superiority of Rhinogen after 6 weeks of treatment. Furthermore, SH cream scored significantly better than nasal ointment H.E.C. with respect to the organoleptic parameters (smelling and sensation of cooling).	Neither adverse reactions were reported nor observed for Rhinogen, whereas three patients in the H.E.C. -treated group complained of sore throat and burning sensation when the ointment flowed down into the pharynx.
Gouteva I. et al.[18]	Prospective, observational parallel group	49 pts divided in SH group (27) and in control group (22)	mean age of the total population was 33.12 ±11.0.	49 patients affected bychronic rhinosinusitis undergoing to FESS	The control group received customary conditioning preparations that were not documented. The nasal spray group received Hysan Pflegespray, a solution containing 0.25mg/mL SH, 2% dexpanthenol. One to two puffs of nasal spray to each nostril were to be administered three times, distributed evenly throughout the day. In both group, treatments were administered for 6 weeks after surgery in the nasal cavity.	Rhino/Endoscopic mucosal findings. (nasal dryness, dried nasalmucus, fibrin deposition, and development of obstructions); Patient evaluation of nasal breathing and foreign body sensation. pre- and pos-toperative. Rhinomanometry (8th–10th Post-operative Day).	Regarding all RSS parameters, mucosal regeneration achieved good final results in both groups, tending to a better improvement through the spray application, without statistically significant differences during the whole assessment period. No statistically significant benefit was identified for nasal breathing, foreign body sensation, and average rhinomanometric volume flow, which improved by 12.31% (control group) and 11.24% (nasal spray group).	
Macchi A. et al. [19]	Prospective, open label, rnd, controlled, parallel group	75 Patients: SH group (38); Control group (37)	SH group (6); Control group (7)	Recurrent upper respiratory tract infections	Selected patients started aerosol therapy with nasal washes. Intermittent treatment was administered twice daily for 15 consecutive days per month, for three consecutive months. Patients in SH group received 9 mg vial of sodium hyaluronate plus 3 ml of saline solution, while patients in control group were only treated with 6 ml of saline solution.	Dichotomic variables: (1) rhinitis (2) post nasal drip (3) nasal dysponea (4) mycetes (5) biofilm. Quantitative variables: (1) ciliary motility (2) adenoid hypertrophy (3) presence of bacteria (4) neutrophils (5) eosinophils (6) Mast cells	Treatment with SH was associated with significant improvements compared to saline solution alone for rhinitis, nasal dyspnoea and biofilm. The results showed significant differences which favoured SH group for ciliary motility, adenoid hypertrophy, presence of bacteria and neutrophilis.	
Di Cicco M et al.[20]	Prospective, double blind, rnd, controlled, parallel group	27 Patients: SH group (14); Control group(13)	SH + tobramycin group (16); SH group (15)	Patients affetced by cystic fibrosis and nasal infection (pseudomonas aeruginosa and/or staphylococcus aureus)	The test nasal spray formulation consisted of a 10-mL aqueous solution containing 0.2% SH and 3% tobramycin sulphate. Control nasal spray formulation was identical in presentation but contained only 0.2% SH. Each patient nebulized 100 μL of the assigned product into each nostril 3 times a day for 14 days.	Endoscopic evaluation (eutrophic, hyperemic, dystrophic nasal mucosa, mucopurulent secretion, hypertrophy of the left and right turbinate). Symptoms of nasal obstruction (mucopurulent rhinorrhea, hyposmia, /anosmia, snoring, headache, /facial pain). Positive pseudomonas aeruginosa and positive staphylococcus aureus (sputum, nasal swab).	Combination of SH and tobramycin reduced hyposmia/anosmia, headache/facial pain, and nasal mucopurulent secretion, and improved the status of the nasal mucosa. More patients in the SH and tobramycin group had negative nasal swab culture for S. aureus at the end of treatment, while all the patients with positive nasal swab culture for P. aeruginosa showed a reduction of bacterial load.	
Gelardi M, et al.[9]	Prospective, blindedl, rnd, controlled, parallel group	78 Patients: SH group (39); Control group (39)	SH group (21–63); Control group (22–61)	Patients affected by allergic and non-allergic vasomotor rhinitis	Treatments were administered for 30 days. SH group: mometasone furoate nasal spray 50 μg/spray, oral desloratadine (5 mg once daily)SH (9mg twice a day aerosolized in 3mL sodium chloride 0.9%)administered 30 minutes after the mometasone. Control group: mometasone furoate,oral desloratadine, sodium chloride 6 mL twice a day aerosolized.	Cytologic outcomes (neutrophils, eosinophils, mast cells, lymphocytes, bacteria spores, infectious stains biofilms); Clinical outcomes (cough, asthma, rhinorrhea, postnasal drip, halitosis, itch, sneezing, nasal congestion);Endoscopic outcomes (edema, hyperemia, deepithelialization,exudate, nasal hyperactivity).	Significant improvement in nasal neutrophil cytology scores was seen in patients receiving SH. The addition of SH to mometasone furoate and desloratadine significantly improved sneezing, rhinorrhea, and nasal obstruction at 1 month. A significant improvement in exudate was observed in SH recipients compared to those in the control group at 1 month.	
Cassandro E, et al.[10]	Prospective,open label, rnd, controlled, parallel group	80 Patients: 4 treatment groups of 20 pts	group 1 (38); group 2 (38); group 3 (39); group 4 (39)	Patients affected by CRS and nasal polyps undergoing FESS	**group1)** nebulized saline aerosl therapy bid; **group2)** intranasal corticosteroid spray mometasone furoate nasal spray 200 ¼g dbid; **group3)** nebulized SH: aerosol therapy with 3ml SH 9mg and 2 saline bid; **group4)** intranasal corticosteroid spray + nebulized SH. The treatment period was 3 months.	Nasal symptom score, endoscopic apparence score, radiologic score, rhinomanometry and saccharine clearence tests before, during and after the treatment period.	Significant improvements in nasal symptom score, endoscopic apparence score, radiologic score, rhinomanometry and saccharine clearence test were observed in the nSH, ICS and in ICS+nSH group after 1 month of treatment, these effect were also seen after 3 months after treatment. The treatment ICS + nSH appeared to be the most effective therapy, reaching a statistically improvments in the control of nasal symptoms.	
Modrzyński M.[11]	Prospective, open label	3 pts in SH group, no control, group	3 patients (48)	Patients affected by empty nose symdrome and atrophic rhinitis	The patients were locally anesthetized by inserting drains filled with 2% lidocaine and adrenalin. The next step was performed under magnification: a CO2 laser, 2 W, contact mode was applied to make tiny punctures into the mucosa under nasal concha membrane. Next, while withdrawing the needle and under careful inspection, a preparation of 0.3–0.4 mL of SH was administered. SH was also injected to a similar depth on both sides, under the perichondrium of the nasal septum, opposite to bottom nasal concha.	Clinical outcomes, rhinoscopy and acoustic rhinometry outcomes	All patients achieved significant improvements in terms of increased airflow sensation throuh the nose, dryness of secretion, and crusting.	
Casale M, et al [12]	Prospective,open label, rnd, controlled, parallel group	39 Patients: SH group (21); Control group (18)	SH group (44); Control group (38)	Patients affected by chronic rhinosinusitis	SH group received SH (3ml of SH were dissolved in 2 ml of isotonic solution) twice a day for 10 days per months over 3 months by Rinowash. The control group received standard saline solution (5 cc) by Rinowash twice a day for 3 months	CRS questionnaire about main CRS symptoms shown. Endoscopic nasal examination performed by a flexible nasal fiberendoscope. The findings were scored with respect to middle turbinate edema and nasal secretions.	SH significantly improved quality of life in patients with sinonasal symptoms in the CRS questionnaire score, unlike control group that showed post-treatment CRS questionnaires similar to the pre-treatment questionnaire. Both the reduction of mucosal edema of the middle turbinate and the reduction of nasal secretions were statistically significant in the SH group.	
JH Lee et al.[13]	Prospective, open label	33 Patients in SH group, no control group	SH group (40)	Patients with recurrent oral ulcers (17 Behcet's disease, 16 recurrent aphthous ulcerations)	0.2% SH gel twice a day for 2 weeks.	Subjective assessment: number of ulcers, healing period and VAS; Objective assessment: number and maximal size of ulcer.	A subjective reduction in the number of ulcers was reported by 72.7% of the patients. A decrease in the ulcer healing period was reported by 72.7%of the patients; 75.8% of patients experienced improvement in VAS for pain. Objective inspection of the ulcers showed a reduction of numbers in 57.6% of the patients, and 78.8% of the ulcers showed a decrease in area. Among the inflammatory signs, swelling and local heat were significantly improved after treatment.	
A.Nolan et al. [14]	Prospective,double blind, rnd, controlled, parallel group	120 Patients: SH group (60); Control group (56)	SH group (37); Control group (36)	Patients with recurrent aphthous ulcerations	For these patients a topical application of SH gel 0.2% or placebo was applied by a Clinician. Patients were instructed how to apply gel for subsequent applications. After the first 60 min, patients were given a sufficient supply of gel to apply two to three times per day for the next 7 days.	Mean number of ulcers; ulcer history during 7-day investigation period; number of patients with ulcer occurrence during 7-day investigation period; distribution of scores from patients overall assessment of their treatment (very good, good, moderate, poor, very poor, not recorded)	Patients in both groups reported a rapid reduction in their discomfort scores arising for their ulcers. This level of reduction was sustained for both treatment groups for about 30 min. There after scores started to return to baseline. There was a slight decline in the number of ulcers, irrespective of treatments over the 7 day. On day 5 patients in the SH group had significantly fewer ulcers than those treated with placebo. In both treatment groups, new ulcers occurred throughout the investigation period but on day 4 the incidence of new ulcer occurrence was significantly lower in the SH group.	
Barber C. et al. [15]	Prospective, open label, rnd, controlled, parallel group	20 Patients: Gelclair group (10); Control group (10)	Gelclair group (64); Control group (59)	Patients with radiotherapy-induced oral mucositis	Patients in both groups were instructed to use their medication four times during the 24-h period, 30 min before meals. Those randomised to Gelclair were told to stir well and immediately swish and gargle for at least 1 min. Those randomised to the Sucralfate and Mucaine (control group) were instructed to swish 10 ml of Sucralfate around the mouth for at least 1 min.	Primary outcome measures: general pain and pain on speaking VAS. Secondary outcome measures: swallowing assessment (self-recording swallowing scales: saliva only, liquids only, soft foods only, can eat anything but hard). Compliance	No relevant observations with regard to general pain, pain on speaking, or ability to eat and drink, were observed for patients with higher grades of OM (i.e. grades III and IV).	Only 1 patient reported mild inflammation and stinging in the oral cavity using Gelclair
Palmieri B et al. [16]	Prospective,double blind, cross over, pbo controlled	20 pts divided in 2 groups according to c.o. design, receiving SH +CS or placebo	SH+CS/Pbo group (55)	Patients with heartburn and/or acid regurgitation	Patients were randomized to one of the two treatment periods and received the first study drug for 14 days. Patients could received either one spoon of syrup containing SH + CS or placebo, administered, far from meals, every 8 hours during daytime and two spoons at bedtime. After a wash out period, patients received either the drug or placebo for a second period of 14 days.	**1** ) Sum of Symptoms Score Intensity(SSSI), 2) Symptoms Score Intensity Difference (SSID), 3) Heartburn and acid regurgitation score intensity difference, 4) Complete symptom disappearance, 5) Rate of patients reporting good speed of action.	At the end of SH + CS treatment both SSSI and SSID were significantly lower as a result of significant changes in terms of heartburn intensity and acid regurgitation intensity. Symptoms complete disappearance was higher after SH+CS treatment and the time to disappearance of symptoms was significantly shorter. An higher, percentage of patients reporting good speed of action.	

### SH and mucosal wound healing after nasal and sinus surgery

Macchi et al. showed that intermittent treatment with SH solution through nasal washes (9 mg vial of SH plus 3 ml saline/daily given for 15 days per months over 3 months) following functional endoscopy sinus surgery (FESS) was associated with significant improvements in symptoms, endoscopic appearance (reduction of nasal edema, crusting and secretions) and ciliary motility, compared to saline nasal washes [15]. Casale et al. examined the potential role of SH (9mg of SH plus 2 ml of sodium chloride 0,9% twice a day for 14 days, trough nasal washes, starting on the first day after surgery) in the radiofrequency surgery that was performed for chronic inferior turbinate hypertrophy, showing a significantly more rapid recovery after surgery especially in the first 2 weeks, with a strong impact on quality of life of these patients in the first weeks after surgery [16]. Gelardi et al. proved the SH efficacy both in clinical/endoscopic parameters and in mucociliary transport in patients undergoing FESS for nasal polyposis (NP)[17], with nasal lavages based on 9 mg of SH nebulized in 3 ml sodium chloride 0,9% twice a day for 30 days, from the second day after surgery. Cantone et al. obtained a faster improvement both in general health and in specific sinonasal status through the use of topical SH, compared to control group only treated with saline solution [18]. Also Soldati et al. achieved a faster mucosal wound healing after nasal and sinus surgery using Rhinogen, a new marketed nasal cream containing hyaluronic acid [19]. The same results both in clinical and functional nasal parameters were obtained, by Gouteva et al. after nasal surgery, with a spray containing SH and Dexpanthenol Hysan Pflegespray (HP)[20].

### SH and chronic upper airway inflammation in pediatric population

Macchi et al. demonstrated that intermittent SH treatment with nasal washes was useful in pediatric patients suffering from recurrent upper respiratory tract infections; in particular, the SH group had a significant improvement in ciliary motility, cytological, microbiological, endoscopic and clinical outcomes compared to saline group. The results also showed a significant adenoid size reduction and a lower presence of bacteria and neutrophils, both assessed through direct evaluation with optical microscopy [21]. Di Cicco et al. reported that SH in association with tobramycin was more effective than hyaluronate alone for the treatment of bacterial rhinosinusitis in patients affected by cystic fibrosis (CF), reducing both upper airway symptoms (hyposmia/anosmia and headache/facial pain) and muco-purulent secretions [22].

### SH in adult population

Gelardi et al. noted that in patients with allergic and non allergic rhinitis the addition of SH to intranasal corticosteroids and systemic antihistamines achieved a significant reduction of the neutrophil count observed on nasal cytology with an improvement of the main nasal symptoms and endoscopic parameters compared to what obtained in patients treated with intranasal corticosteroids and systemic antihistamines without SH [23]. Cassandro et al. showed that in patients with chronic respiratory syndrome (CRS) and nasal polyposis (NP) the addition of nebulized SH to intranasal corticosteroid spray, produced a rapid and significant improvement in nasal symptom score, endoscopic parameters score, radiologic score, rhinomanometry and saccharine clearance test with respect to control group [24].

An interesting study performed by Modrzyński on empty nose syndrome (ENS) and atrophic rhinitis, pointed out that submucosal injections of SH into the inferior nasal concha and under the mucous membrane of the septum, achieved both a significant improvement of nasal airflow sensation, and less severe dryness of secretion and crusting [25]. Casale et al., in order to minimize symptoms and prevent exacerbation of CRS tested an alternative therapeutic option to nasal douches with saline solution after FESS; they investigated and proved that intermittent treatment with topical 9 mg SH through nasal washes (during 10 days per month, over 3 months during winter months) both significantly improved quality of life in CRS patients with sino-nasal symptoms and reduced mucosal edema of the middle turbinate and nasal secretions, compared to what observed in patients only treated with saline solution [27].

### SH in upper digestive airways

Recent data from literature highlight the use of SH as topical treatment for oral chronic inflammation, as well; in particular some authors reported that topical application of 0.2% SH gel twice daily for 2 weeks seems to be an effective and safe therapy in patients with recurrent aphthous ulcers (RAU)[27] and oral ulcers of Behçet’s disease (BD)[7].

Barber C. et al. investigated in this preliminary study the effect of SH on radiotherapy induced oral mucositis (OM) and found that the use of Gelclair (SH-four times during the 24-h period, 30 min before meals) is no more effective than current standard practice (Sucralfate and Mucaine) in relieving general pain, pain on speaking, ability to eat and drink, especially for the most severe cases of OM [28]

In the last study we examined in our present review, Palmieri B et al. were the first to evaluate the potential role of SH in adults affected by gastro-esophageal reflux disease (GERD), showing that a treatment with a fixed oral combination of SH and chondroitin-sulphate for 14 days, produced a fast relief of GERD symptoms in adults with symptoms of non-erosive GERD and a low response to PPIs [29].

## Discussion

Hyaluronan is a non-sulfated glycosaminoglycan found in the extracellular matrix of all vertebrate tissues, which plays a multifunctional role in scar-free wound healing, while also being essential in the homeostasis of physiology in UADT^5,19)^. Literature data emerged from our analysis allow us to affirm that topical SH can be useful as coadjutant treatment in the post-operative period both for nasal and sinus surgery as a supportive treatment for a faster improvement of nasal breathing and to reduce patients' discomfort in the postoperative phase [15–19].

Despite the advances in surgical technique, poor wound healing after endoscopic sinus and nasal surgery still represents a significant problem, indeed. Due to the proximity of denuded surfaces and the presence of clot, the restructured sinus anatomy is prone to subsequent development of synechiae, ostial stenosis, extensive crusts, scar contraction, impairment of ventilation of the para-nasal sinuses due to the presence of secretions, oedema, bleeding, thus frustrating the good results obtained from a well-performed surgical procedure [20]. In order to solve these problems, otolaryngologists have proposed several different solutions although not uniformly driven by evidence-based outcome research: the insertion of physical barriers, such as packing and stents (absorbable and non absorbable); repeated postoperative debridement and irrigation to remove eschars, crusts, and nascent synechiae; modifications of surgical technique, such as resection and medial adhesion of the middle turbinate; postoperative systemic or topical corticosteroids to discourage the development of granulation and scar tissue. Anyway, while these measures haven’t often a significant impact in wound healing, a foreign body stent has even the potential to exacerbate. For the above mentioned reasons and given the accessibility of the sinus cavities after endoscopic sinus surgery (ESS), topical therapies are preferred for all patients [30]. Due to its positive action on scar-free wound healing and on the homeostasis of upper airways [19], topical administration of SH following FESS adds further benefits over saline solution given alone. Interesting results show potential benefits of SH when given to not-operated CRS patients as preventive therapy to minimize symptoms and prevent from exacerbations of chronic rhino-sinusitis [19]. Notably, SH was proposed in patients affected by empty nose syndrome (ENS) along with atrophic rhinitis. Both of these clinical conditions are characterized by excessive widening of the nasal meatus and excessive patency, so patients complain about chronic dryness of the nose, dyspnea sensation, headache, and depressive states. The treatment of ENS and the resulting rhinitis is a complicated clinical problem that is difficult to solve [26]. As far as conservative treatment is concerned, several different strategies are recommended such as moisturization of the mucous membrane (drops, gels, and/or inhalations) whereas current surgical therapeutic options give conflicting results. SH, unlike other materials, exhibits better water absorption. This might be an additional advantage for patients suffering from ENS and atrophic rhinitis because it contributes to better moisturize the mucous membrane [25].

It should also be stressed that in pediatric population, SH has been used for recurrent upper respiratory tract infections and, associated with tobramycin, in patients with CF, proving to be effective in both of these clinical conditions by acting on nasal muco-ciliary clearance and on nasal muco-ciliary defense against injury induced by bacterial products [21,25].

Last but not least, it worths pointing out that SH have been used for years in chronic inflammatory mouth disease and recently it was proposed to relief symptoms in patients suffering from GERD, as well [29].

**Fig 2:**
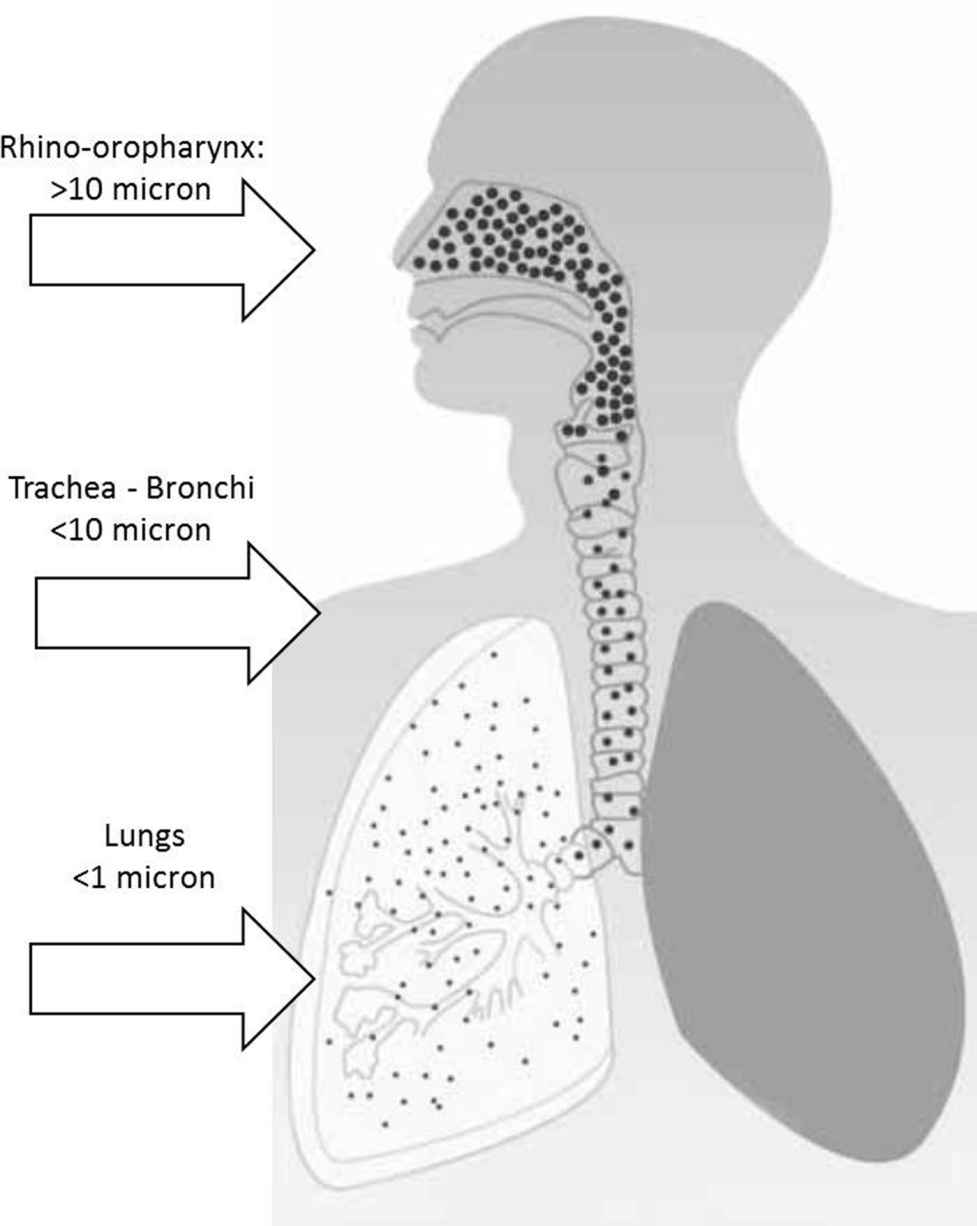
Delivery of drugs to aerodigestive tract.

Finally, it should be emphasize that topical therapies offer an improved ability to deliver high concentrations of pharmacologic agents to the mucosa of the aerodigestive tract for their localized, rather than systemic effects [30]. On this pourpose it worths underlining that while particles of diameter greater to 10 μ have been proved not to generally exceed the rhino-pharyngeal cavity, particles with a diameter less than 10 μ more have been shown to easily reach lower airways, as show in Fig 2.

## Conclusion

Today SH is widely used in many branches of medicine and the potential applications of SH in the chronic inflammatory upper aero digestive tract are various.

Data deriving from the present review of 16 clinical studies demonstrate that, due to its positive action on tissue repair and wound healing, topical administration of SH plays a pivot role in the postoperative phase of patients undergoing FESS, and positive results are generally observed in all the patients suffering from UADT, with a significant improvement of their quality of life.

The strong connection between particle diameter and site of the high concentration of nebulized particles in the upper aero-digestive tract suggests that it should be mandatory to carefully chose the nebulizer device to get better therapeutic results [30]. However, further studies on larger populations are needed to confirm these encouraging results.

## Supporting Information

S1 List of Excluded Studies List of excluded studies ordered according to the reason for which they were excluded. (DOC)

S1 PRISMA Checklist (DOC)
