# Supplementary material for: Hyaluronic Acid: Perspectives in Upper Aero-Digestive Tract. A Systematic Review
Source: PLoS One. 2015 Jun 29;10(6):e0130637. doi: 10.1371/journal.pone.0130637 (PMC4487693; doi:10.1371/journal.pone.0130637)
Supplement: S1 List of Excluded Studies — (DOC) [file pone.0130637.s001.doc]

We exclude:

- Studies based on animal models (n=7)

1) Proctor M1, Proctor K, Shu XZ, McGill LD, Prestwich GD, Orlandi RR. Composition of hyaluronan affects wound healing in the rabbit maxillary sinus. Am J Rhinol. 2006 Mar-Apr;20(2):206-11.

2) Luo Y1, Kobler JB, Heaton JT, Jia X, Zeitels SM, Langer R. Injectable hyaluronic acid-dextran hydrogels and effects of implantation in ferret vocal fold. J Biomed Mater Res B Appl Biomater. 2010 May;93(2):386-93.

3) Choi JS1, Kim NJ, Klemuk S, Jang YH, Park IS, Ahn KH, Lim JY, Kim YM. Preservation of viscoelastic properties of rabbit vocal folds after implantation of hyaluronic Acid-based biomaterials. Otolaryngol Head Neck Surg. 2012 Sep;147(3):515-21.

4) Borzacchiello A, Mayol L, Gärskog O, Dahlqvist A, Ambrosio L. Evaluation of injection augmentation treatment of hyaluronic acid based materials on rabbit vocal folds viscoelasticity. J Mater Sci Mater Med. 2005 Jun;16(6):553-7.

5) Robinson S, Adams D, Wormald PJ. The effect of nasal packing and prednisolone on mucosal healing and reciliation in a sheep model. Rhinology. 2004 Jun;42(2):68-72.

6) Rajapaksa S, McIntosh D, Cowin A, Adams D, Wormald PJ. The effect of insulin-like growth factor 1 incorporated into a hyaluronic acid-based nasal pack on nasal mucosal healing in a healthy sheep model and a sheep model of chronic sinusitis. Am J Rhinol. 2005 May-Jun;19(3):251-6.

7) Chen Q, Sun G, Wang Y, Zhong W, Shu XZ. The evaluation of two new hyaluronan hydrogels as nasal dressing in the rabbit maxillary sinus. Am J Rhinol Allergy. 2012 Mar-Apr;26(2):152-6.

- studies based on the use of HA with nasal packing (n= 4)

1) Chang C, Hong SM, Cho JH, Shim SY, Cho JS, Lee HM. A Randomized, Multi-Center, Single Blind, Active-Controlled, Matched Pairs Clinical Study to Evaluate Prevention of Adhesion Formation and Safety of HyFence in Patients After Endoscopic Sinus Surgery.

Clin Exp Otorhinolaryngol. 2014 Mar;7(1):30-5

2) Berlucchi M, Castelnuovo P, Vincenzi A, Morra B, Pasquini E. Endoscopic outcomes of resorbable nasal packing after functional endoscopic sinus surgery: a multicenter prospective randomized controlled study. Eur Arch Otorhinolaryngol. 2009 Jun;266(6):839-45.

3) Song KJ, Lee HM, Lee EJ, Kwon JH, Jo KH, Kim KS. Anti-adhesive effect of a thermosensitive poloxamer applied after the removal of nasal packing in endoscopic sinus surgery: a randomised multicentre clinical trial. Clin Otolaryngol. 2013 Jun;38(3):225-30.

4) Franklin JH1, Wright ED. Randomized, controlled, study of absorbable nasal packing on outcomes of surgical treatment of rhinosinusitis with polyposis. Am J Rhinol. 2007 Mar-Apr;21(2):214-7.

- studies based on the use of HA with nasal dressing (n=2)

1) Woodworth BA, Chandra RK, Hoy MJ, Lee FS, Schlosser RJ, Gillespie MB. Randomized controlled trial of hyaluronic acid/carboxymethylcellulose dressing after endoscopic sinus surgery. ORL J Otorhinolaryngol Relat Spec. 2010;72(2):101-5.

2) Shi R1, Zhou J, Wang B, Wu Q, Shen Y, Wang P, Wang J, Wang Y, Chen Y, Shu XZ. The clinical outcomes of new hyaluronan nasal dressing: a prospective, randomized, controlled study.Am J Rhinol Allergy. 2013 Jan;27(1):71-6.

- Studies with an invasive administration of hyaluronic acid (n=10)

1. Hertegård S, Hallén L, Laurent C, Lindström E, Olofsson K, Testad P, Dahlqvist A. Cross-linked hyaluronan versus collagen for injection treatment of glottal insufficiency: 2-year follow-up. Acta Otolaryngol. 2004 Dec;124(10):1208-14.
2. Shamanna SG, Bosch JD. Injection laryngoplasty: a serious reaction to hyaluronic acid. J Otolaryngol Head Neck Surg. 2011 Oct;40(5):E39-42.
3. Upton DC, Johnson M, Zelazny SK, Dailey SH. Prospective evaluation of office-based injection laryngoplasty with hyaluronic acid gel. Ann Otol Rhinol Laryngol. 2013 Sep;122(9):541-6.
4. Szkiełkowska A, Miaśkiewicz B, Remacle M, Krasnodębska P, Skarżyński H. Quality of the voice after injection of hyaluronic acid into the vocal fold. Med Sci Monit. 2013 Apr 17;19:276-82.
5. Reiter R, Brosch S. Laryngoplasty with hyaluronic acid in patients with unilateral vocal fold paralysis. J Voice. 2012 Nov;26(6):785-91.
6. Wen MH, Cheng PW, Liao LJ, Chou HW, Wang CT. Treatment outcomes of injection laryngoplasty using cross-linked porcine collagen and hyaluronic acid. Otolaryngol Head Neck Surg. 2013 Dec;149(6):900-6.
7. Finck CL, Harmegnies B, Remacle A, Lefebvre P. Implantation of esterified hyaluronic acid in microdissected Reinke's space after vocal fold microsurgery: short- and long-term results. J Voice. 2010 Sep;24(5):626-35.
8. Hertegård S, Hallén L, Laurent C, Lindström E, Olofsson K, Testad P, Dahlqvist A. Cross-linked hyaluronan used as augmentation substance for treatment of glottal insufficiency: safety aspects and vocal fold function. Laryngoscope. 2002 Dec;112(12):2211-9.
9. Matheny KE, Tseng EY, Carter KB Jr, Cobb WB, Fong KJ. Self-cross-linked hyaluronic acid hydrogel in ethmoidectomy: a randomized, controlled trial. Am J Rhinol Allergy. 2014 Nov-Dec;28(6):508-13. doi: 10.2500/ajra.2014.28.4106. Epub 2014 Sep 11.
10. Ahn JH, Shim MJ. The use of sodium hyaluronate-carboxymethylcellulose to prevent postoperative mastication pain from harvesting of temporalis fascia. Auris Nasus Larynx. 2013 Feb;40(1):7-10.
